# Credibilistic Mean-Semi-Entropy Model for Multi-Period Portfolio Selection with Background Risk

**DOI:** 10.3390/e21100944

**Published:** 2019-09-26

**Authors:** Jun Zhang, Qian Li

**Affiliations:** School of Management and Engineering, Capital University of Economics and Business, Beijing 100010, China; zhangjun@cueb.edu.cn

**Keywords:** background risk, fuzzy semi-entropy, multi-period portfolio selection, dragonfly algorithm, credibility theory

## Abstract

In financial markets, investors will face not only portfolio risk but also background risk. This paper proposes a credibilistic multi-objective mean-semi-entropy model with background risk for multi-period portfolio selection. In addition, realistic constraints such as liquidity, cardinality constraints, transaction costs, and buy-in thresholds are considered. For solving the proposed multi-objective problem efficiently, a novel hybrid algorithm named Hybrid Dragonfly Algorithm-Genetic Algorithm (HDA-GA) is designed by combining the advantages of the dragonfly algorithm (DA) and non-dominated sorting genetic algorithm II (NSGA II). Moreover, in the hybrid algorithm, parameter optimization, constraints handling, and external archive approaches are used to improve the ability of finding accurate approximations of Pareto optimal solutions with high diversity and coverage. Finally, we provide several empirical studies to show the validity of the proposed approaches.

## 1. Introduction

As a research field, portfolio selection is used to accomplish the investments in financial markets by spreading investors’ capital among several different assets considering return and risk. Since the pioneering work of Markowitz [1] in single-period investment problems, the mean–variance portfolio selection problem has attracted much attention and has become a research hotspot. By introducing different risk measures, a large variety of portfolio selection models have been presented, such as the mean–variance–skewness model [2], the mean-conditional value at risk (CVaR) model [3], the mean-value at risk (VaR) model [4], the mean-semi-variance model [5] and the minimax risk model [6]. In addition, entropy can also be used as a risk measure because it does not depend on symmetric membership functions and can be calculated from non-metric data. Philippatos and Wilson [7] first replaced variance with entropy as a risk measure. Later, Rödder et al.  [8] provided a new and efficient method for determining the portfolio weights on the basis of a rule inference mechanism with both maximum entropy and minimum relative entropy. Nawrocki and Harding [9] provided two alternative weighted computations of entropy to measure portfolio risk. Usta and Kantar [10] presented a multi-objective model founded on mean, variance, skewness and entropy to adequately diversify the portfolio. Yu et al. [11] discussed the performance of the models with diverse entropy measures by comparing the mean–variance efficiency, portfolio values, and diversity.

Traditionally, researchers dealt with the uncertainty of portfolio selection problems by applying probability theory. For example, Beraldi et al. [12] proposed a mean-CVaR model considering a complex transaction cost structure, and designed a specialized Branch and Bound method to solve the proposed model. Huang [13] built a new type of model based on a risk curve. However, many non-probabilistic elements, such as economics, politics and social circumstances, exist in real capital markets and affect investment decisions. With the introduction of fuzzy set theory  [14], an increasing number of scholars began to investigate the portfolio selection problems in the fuzzy environment. Assuming that the returns are fuzzy, there exist numerous papers employing possibility theory for fuzzy portfolio selections; see, for example, Vercher et al. [15], Chen [16], Jana et al. [17], Chen and Tsaur [18], Liu and Zhang [19], and Chen and Xu [20]. Although possibility theory is widely used, it has limitations. For instance, it is not self-dual. To overcome this drawback, Liu [21] proposed credibility theory. Under the framework of the credibility theory, Gupta et al. [22] presented a multi-objective expected value model using risk, liquidity, short-term return, and long-term return. Gupta et al. [23] proposed a multi-criteria credibilistic portfolio rebalancing model considering portfolio risk as a risk curve. Liu et al. [24] built a class of credibilistic mean-CVaR portfolio optimization models. Huang [25] provided two credibility-based portfolio selection models according to two types of chance criteria. Li et al. [26] discussed a maximum likelihood estimation and a minimum entropy estimation for expected value and variance of normal fuzzy numbers in fuzzy portfolio selection. Jalota et al.  [27] modeled return, illiquidity, and risk of different kinds of assets by using L-R fuzzy numbers in a credibilistic framework. Deng et al. [28] built a mean-entropy model in the framework of credibility theory. Xu et al. [29] proposed a credibilistic semi-variance project portfolio model with skewness risk constraints.

In reality, except for portfolio risk, investors frequently face background risks such as losses of human capital, pensions, unexpected health-related costs, labor incomes, and real estate investments. Therefore, an increasing number of scholars have studied portfolio selection problems with background risk. Alghalith [30] introduced a dynamic investment model to illustrate the impact of background risk and found a negative correlation between the background risk and portfolio risk. Huang and Wang [31] analyzed the characteristics of the portfolio with background risk under a mean–variance framework. Jiang et al. [32] discussed the influence of background risk in the framework of the mean–variance model. Biptista [33] proposed a mean–variance model considering background risk and analyzed the circumstances under which investors can optimally entrust the portfolio managers to administer their wealth. Biptista [34] introduced mental accounts as well as background risk into portfolio selection and derived the efficient portfolio frontier. In addition to the above studies, few researchers considered background risk in fuzzy portfolio selection problems. Thus, to the best of our knowledge, the only exceptions are the following two studies. Xu et al. [35] provided a fuzzy portfolio selection model taking the vagueness of the investors’ performances and background risk into account. Li et al.  [36] gave a possibility-based portfolio selection model considering background risk.

All of the previous literature is in the framework of single-period. However, investment is a long-term process, and investors need to redistribute their funds over time. Numerous scholars have studied portfolio selection problems from single-period to multi-period cases. Some representative works on multi-period portfolio selections include Chen et al. [37], Zhang et al. [38], Liagkouras and Metaxiotis [39], Li et al. [40], and Zhang et al. [41]. On the other hand, several researchers have researched multi-period portfolio selection problems based on credibility theory. Typically, Mehlawat  [42] developed credibility-based multi-objective models taking multi-choice aspiration levels into consideration for multi-period portfolio optimization problems. Mohebei and Najafi [43] presented a multi-period mean-VaR model by combining the credibility theory with a scenario tree. Liu et al.  [44] designed a credibilistic multi-period mean-LAD-entropy model considering bankruptcy control and bound constraints. Zhang and Liu [45] gave a credibility-based model with a bankruptcy risk control constraint for solving multi-period portfolio selection problems. Guo et al.  [46] formulated a multi-period credibilistic mean–variance model with the terminal return constraint and V-shaped transaction cost.

In recent years, swarm intelligence-based optimization techniques have attracted increased attention. A literature review reveals the effectiveness of swarm intelligence algorithms in solving complex optimization problems, such as the salp swarm algorithm (SSA) [47], the artificial bee colony algorithm (ABC) [48], the firefly algorithm (FA) [49], and the particle swarm optimization (PSO) [50]. The dragonfly algorithm (DA) is a fairly novel swarm intelligence optimization technique proposed by Mirjalili [51] and is based on the static and dynamic swarming behaviors of dragonflies in nature. Compared with the non-dominated sorting genetic algorithm II (NSGA II) and PSO, DA has advantages in dealing with optimization problems and has been applied in many fields. Recently, Mirjalili [51] proposed a multi-objective dragonfly algorithm (MODA) and applied it to submarine propeller optimization problems. Amroune et al.  [52] used a hybrid dragonfly optimization algorithm and support vector regression to solve a power system voltage stability assessment problem. Suresh and Sreejith  [53] used the dragonfly algorithm to solve static economic dispatch with solar energy. Mafarja et al.  [54] presented a variety of S-shaped and V-shaped transfer functions to balance the exploration and exploitation in the binary dragonfly algorithm. Khadanga et al.  [55] proposed a hybrid dragonfly and pattern search algorithm approach and used it in tilt integral derivative controller design. Ghanem and Jantan [56] combined ABC and DA to train a multi-layer perceptron. Sree and Murugan  [57] developed a memory-based hybrid dragonfly algorithm with the concept of PSO gbest and pbest for solving three engineering design problems.

Although numerous studies have been performed for multi-period fuzzy portfolio selections, few studies have considered background risk under the framework of credibility theory. Moreover, to date, the application of the DA algorithm in portfolio selection problems is relatively rare. The purpose of this paper is to investigate the multi-period portfolio selection problem with background risk in the framework of credibility theory. The main contributions of this paper are as follows: (1) We formulate a credibility-based mean-semi-entropy multi-period portfolio model, considering background risk and several constraints, namely cardinality, liquidity, and buy-in thresholds; (2) We develop a new meta-heuristic approach, combining the strengths of DA and NSGA II. In the proposed algorithm, parameter optimization, constraints handling, and external archive approaches are proposed to improve the ability of finding accurate approximations of Pareto optimal solutions with high diversity and coverage; (3) We run several experiments based on ZDT benchmark functions and a real-world empirical application to verify the effectiveness of the proposed methods.

The rest of this paper is organized as follows: Section 2 describes the preliminaries. In Section 3, we build a multi-period credibility-based mean-semi-entropy model considering background risk. Section 4 discusses the solution method and proposes a hybrid algorithm. In Section 5, numerical experiments are examined to verify the validity of the proposed model and the hybrid algorithm. In Section 6, we submit our conclusions.

## 2. Preliminaries

Let Θ be a nonempty set. Assume that *P* is the power set of Θ. Each element in *P* is called an event. In order to present an axiomatic definition of credibility, it is necessary to assign a number Cr{A} to each event *A*. Cr{A} indicates the credibility that the event will happen. Θ has the following mathematical axioms:

**Axiom** **1** (*Normality*)**.** *Cr{Θ} = 1,*

**Axiom** **2** (*Monotonicity*)**.** *Cr{A}≤Cr{B} wherever A⊂B,*

**Axiom** **3** (*Self-Duality*)**.** *$Cr\left\{A\right\}+Cr\left\{A^{c}\right\}=1$ for any event A,*

**Axiom** **4** (*Maximality*)***.**$Cr\left\{U_{i}A_{i}\right\}=sup_{i}Cr\left\{A_{i}\right\}$ for any event $\left\{A_{i}\right\}$ with $sup_{i}Cr\left\{A\right\}<0.5$.*

If the set function Cr satisfies the aforementioned four axioms, the {Θ,P,Cr} will be credibility space.

**Definition** **1.** *Let ξ be a fuzzy variable defined on the credibility space {Θ,P,Cr} with membership function μ{x}. For any set A of real numbers, the credibility is defined as*

(1) $$Cr\left\{\xi \in A\right\}=\frac{1}{2}\left(\underset{x\in A}{\sup}\mu \left(x\right)+1-\underset{x\in A^{c}}{\sup}\mu \left(x\right)\right).$$

Credibility measure is an increasing function of set *A*. It is obvious that the credibility measure is self-dual.

**Definition** **2.** *Let ξ be a fuzzy variable; the expected value of ξ is defined as*

(2) $$E\left[\xi \right]=\int_{0}^{\infty }Cr\left\{\xi \geq r\right\}dr-\int_{-\infty }^{0}Cr\left\{\xi \leq r\right\}dr.$$

**Theorem** **1.** *Let ξ be a fuzzy variable with a finite expected value; let μ and ν be any given two real numbers. Then,*

(3) E[μξ+ν]=μE[ξ]+ν.

**Theorem** **2.** *Suppose that ξ and η are two independent fuzzy variables. The expected value of these variables are finite. Then, for any numbers μ and ν,*

(4) E[μξ+νη]=μE[ξ]+νE[η].

**Example** **1.** *The expected value for the trapezoidal fuzzy variable $\xi =\left(\xi_{a},\xi_{b},\xi_{c},\xi_{d}\right)$ is given by*

(5) $$E\left[\xi \right]=\frac{\xi_{a}+\xi_{b}+\xi_{c}+\xi_{d}}{4}.$$

For the sake of determining the credibility of a fuzzy event, the trapezoidal fuzzy variable ξ has a membership function illustrated below:

(6) $$\begin{array}{c} \mu \left(r\right)=\left\{\begin{array}{cc} \frac{r-\xi_{a}}{\xi_{b}-\xi_{a}}, & \mathrm{if}\;\xi_{a}\leq r\leq \xi_{b},\\ 1, & \mathrm{if}\;\xi_{b}\leq r\leq \xi_{c},\\ \frac{\xi_{d}-r}{\xi_{d}-\xi_{c}},\; & \mathrm{if}\;\xi_{c}\leq r\leq \xi_{d},\\ 0, & \mathrm{otherwise}. \end{array}\right. \end{array}$$

Then, the credibility of fuzzy event {ξ≤r} is given as below:

(7) $$\begin{array}{c} Cr\left\{\xi \leq r\right\}=\left\{\begin{array}{cc} 0, & \mathrm{if}\;r\leq \xi_{a},\\ \frac{r-\xi_{a}}{2(\xi_{b}-\xi_{a})},\; & \mathrm{if}\;\xi_{a}\leq r\leq \xi_{b},\\ \frac{1}{2}, & \mathrm{if}\;\xi_{b}\leq r\leq \xi_{c},\\ \frac{\xi_{d}-2\xi_{c}+r}{2(\xi_{d}-\xi_{c})},\; & \mathrm{if}\;\xi_{c}\leq r\leq \xi_{d},\\ 1, & \mathrm{otherwise}. \end{array}\right. \end{array}$$

## 3. Mean-Semi-Entropy Model for Credibilistic Multi-Period Portfolio Selection

### 3.1. Notation

At the beginning of the investment, we assume that the investor’s initial wealth is $W_{1}$. The investor allocates $W_{1}$ among *n* risky assets and a risk-free asset at the start of T−1 period and acquires the ultimate wealth at the final period *T*. As a matter of convenience, we list all the symbols used below:

*i*: the exponents for the *n* risky assets, i=1,2,…n.
*t*: the exponents for the *T* investment period, t=1,2,…T.
$W_{t}$: the wealth accumulated at the start of the t-th investment period.
$x_{it}$: the proportion of the whole wealth that investor spreads to the i-th risky asset during the t-th investment period.
$r_{it}$: fuzzy variables that represent the return rate on the i-th risky asset during the t-th investment period, $r_{it}=\left(\alpha_{it}^{a},\alpha_{it}^{b},\alpha_{it}^{c},\alpha_{it}^{d}\right)$.
$r_{f}$: the variable that represents the return rate on the risk-free asset.
$r_{b}$: the fuzzy variable that represents the return rate on background asset, $r_{b}=\left(\xi_{a},\xi_{b},\xi_{c},\xi_{d}\right)$.
$ub_{it}$: the upper limit that can be assigned to the i-th risky asset during the t-th investment period.
$lb_{it}$: the lower limit that can be assigned to the i-th risky asset during the t-th investment period.
$f_{it}$: the cost on transaction of the i-th risky asset during the t-th investment period.
$L_{it}$: the fuzzy variables that represent the turnover rates on the i-th risky asset during the t-th investment period, $L_{it}=\left(\beta_{it}^{a},\beta_{it}^{b},\beta_{it}^{c},\beta_{it}^{d}\right)$.
$L_{t}$: the acceptable minimum expected liquidity during the t-th investment period.
$m_{it}$: the 0–1 variables expressing whether the i-th risky asset is chosen for the portfolio during the t-th investment period or not:
  $$\begin{array}{c} m_{it}=\left\{\begin{array}{cc} 1, & \mathrm{if}\;\mathrm{the}\;i\text{-}\text{th}\;\mathrm{risky}\;\mathrm{asset}\;\mathrm{is}\;\mathrm{chosen}\;\mathrm{to}\;\mathrm{the}\;\mathrm{portfolio}\;\mathrm{during}\;\mathrm{the}\;t\text{-}\text{th}\;\mathrm{period},\\ 0, & \mathrm{otherwise}, \end{array}\right. \end{array}$$
$Z_{t}$: the desired number of risky assets that can be chosen for each investment interval.

### 3.2. Objective Functions

#### 3.2.1. Maximize Ultimate Wealth

According to Equations (3) and (5), the expected value of the portfolio $x_{t}$ during the t-th investment period is

(8) $$E\left(\sum_{i=1}^{n}x_{it}r_{it}\right)=\sum_{i=1}^{n}\frac{\alpha_{it}^{a}+\alpha_{it}^{b}+\alpha_{it}^{c}+\alpha_{it}^{d}}{4}x_{it}.$$

Moreover, from Equation (5), the expected value of the background asset is

(9) $$E\left(r_{b}\right)=\frac{\xi_{a}+\xi_{b}+\xi_{c}+\xi_{d}}{4}.$$

Additionally, we apply a V-shaped function that expresses the differences between the two diverse portfolios of the two adjacent periods. Then, the expense on transaction of the i-th risky asset during the t-th investment period is $f_{it}\left|x_{it}-x_{it-1}\right|$. Furthermore, from Equations (8) and (9), the net return rate at period *t* can be denoted as

(10) $$\begin{array}{cc} R_{t}\; & =E\left(\sum_{i=1}^{n}x_{it}r_{it}-f_{t}\right)+\left(1-\sum_{i=1}^{n}x_{it}\right)r_{f}+E\left(r_{b}\right)\\ \; & =\sum_{i=1}^{n}\frac{\alpha_{it}^{a}+\alpha_{it}^{b}+\alpha_{it}^{c}+\alpha_{it}^{d}}{4}x_{it}-\sum_{i=1}^{n}f_{it}\left|x_{it}-x_{it-1}\right|\\ \; & +\frac{\xi_{a}+\xi_{b}+\xi_{c}+\xi_{d}}{4}+\left(1-\sum_{i=1}^{n}x_{it}\right)r_{f}. \end{array}$$

Then, the expected value of the wealth at the beginning of the period t+1 is expressed as $W_{t+1}=W_{t}\left(1+R_{t}\right)$. Thus, after accomplishing the investment through the entirety of investment periods, from Equation (10), the ultimate wealth at the end of the period *T* is denoted as

(11) $$\begin{array}{cc} W_{T+1} & =W_{1}\prod_{t=1}^{T}\left(1+R_{t}\right)\\ \; & =W_{1}\prod_{t=1}^{T}\left(1+\sum_{i=1}^{n}\frac{\alpha_{it}^{a}+\alpha_{it}^{b}+\alpha_{it}^{c}+\alpha_{it}^{d}}{4}x_{it}-\sum_{i=1}^{n}f_{it}\left|x_{it}-x_{it-1}\right|\right.\\ \; & \left.+\frac{\xi_{a}+\xi_{b}+\xi_{c}+\xi_{d}}{4}+\left(1-\sum_{i=1}^{n}x_{it}\right)r_{f}\right). \end{array}$$

#### 3.2.2. Minimize Risk

Fuzzy entropy has been extensively applied to characterize uncertainty since Luca and Termini [58] first defined a non-probabilistic entropy in the framework of fuzzy set entropy. Since then, various definitions for fuzzy entropy have been proposed; see, for example, Li and Liu [59], Zhou et al.  [60], Qin et al. [61], and  Xu et al. [62]. Fuzzy entropy is more convenient than fuzzy variance because it does not depend on symmetric membership functions and can be calculated from non-metric data. It is used to express the uncertainty of both low and high extreme returns. However, what investors really dislike is the downside uncertainty. Therefore, fuzzy semi-entropy introduced by Zhou et al. [60] matches reality more exactly as the downside risk measure. In this section, we used the semi-entropy to quantify the portfolio downside risk.

**Definition** **3.** *Assume that there is a continuous fuzzy variable δ whose expected value E[δ] is finite. The function o(x) is equal to Cr{δ=x}. Then, the  semi-entropy of δ is defined as [60]*

(12) $$S_{e}\left[\delta \right]=\int_{-\infty }^{+\infty }S\left(o{\left(x\right)}^{-}\right)dx,$$

*where $S\left(t\right)=-t\ln t-\left(1-t\right)\ln\left(1-t\right)$ and*

(13) $$o{\left(x_{i}\right)}^{-}=\left\{\begin{array}{cc} o\left(x_{i}\right), & \mathrm{if}\;x_{i}\leq e,\\ 0, & \mathrm{otherwise}. \end{array}\right.$$

Because of $S_{e}\left(0\right)=0$, the semi-entropy of δ can be transformed into

(14) $$S_{e}\left[\delta \right]=\int_{-\infty }^{E[\xi ]}S\left(o\left(x\right)\right)dx.$$

**Theorem** **3.** *Suppose there is a continuous fuzzy variable δ whose expected value E[δ] is finite. Then, for these two real numbers λ and ω with λ>0,*

(15) $$S_{e}\left[\lambda \delta +\omega \right]=\lambda S_{e}\left[\delta \right].$$

**Example** **2.** *Suppose δ is a fuzzy trapezoidal variable with $\delta =\left(\delta_{a},\delta_{b},\delta_{c},\delta_{d}\right)$ whose expected value $E\left[\delta \right]=\left(\delta_{a}+\delta_{b}+\delta_{c}+\delta_{d}\right)/4$. Then, the semi-entropy*

(16) $$S_{e}\left[\delta \right]=\left\{\begin{array}{cc} \left(\delta_{b}-\delta_{a}\right)\rho -\zeta \left(\rho \right),\; & \mathrm{if}\;E\left[\delta \right]\leq \delta_{b},\;\\ \frac{\delta_{b}-\delta_{a}}{2}+\frac{(\delta_{a}+\delta_{c}+\delta_{d}-3\delta_{b})\ln2}{4}, & \mathrm{if}\;\delta_{b}<E\left[\delta \right]\leq \delta_{c},\;\\ \frac{\left(\delta_{b}-\delta_{a}\right)}{2}+\left(\delta_{c}-\delta_{b}\right)\ln2+\zeta \left(\tau \right), & \mathrm{otherwise}, \end{array}\right.\;$$

*where $\rho =\left(\delta_{b}+\delta_{c}+\delta_{d}-3\delta_{a}\right)/8\left(\delta_{b}-\delta_{a}\right)$, $\tau =\left(3\delta_{d}-\delta_{a}-\delta_{b}-\delta_{c}\right)/8\left(\delta_{d}-\delta_{c}\right)$, and $\zeta \left(\chi \right)=\chi^{2}\ln\chi -{\left(1-\chi \right)}^{2}\ln\left(1-\chi \right)$.*

Furthermore, according to Equation (16), we obtain the cumulative portfolio risk with background risk as follows:

(17) $$S_{e}=\sum_{t=1}^{T}\left[s_{e}\left(\sum_{i=1}^{n}x_{it}r_{it}\right)+s_{e}\left(r_{b}\right)\right]$$

In Equation (17), according to the definition of semi-entropy and Equation (16),

(18) $$s_{e}\left(r_{it}\right)=\left\{\begin{array}{cc} \left(b_{it}-a_{it}\right)\rho_{it}-\zeta \left(\rho_{it}\right), & \mathrm{if}\;E\left(r_{it}\right)\leq b_{it},\;\\ \frac{b_{it}-a_{it}}{2}+\frac{(a_{it}+c_{it}+d_{it}-3b_{it})\ln2}{4}, & \mathrm{if}\;b_{it}<E\left(r_{it}\right)\leq c_{it},\\ \frac{b_{it}-a_{it}}{2}+\left(c_{it}-b_{it}\right)\ln2+\zeta \left(\tau_{it}\right), & \mathrm{otherwise}, \end{array}\right.\;$$

where $\rho_{it}=\left(b_{it}+c_{it}+d_{it}-3a_{it}\right)/8\left(b_{it}-a_{it}\right)$, $\tau_{it}=\left(3d_{it}-a_{it}-b_{it}-c_{it}\right)/8\left(d_{it}-c_{it}\right)$, and $\zeta \left(\chi \right)=\chi^{2}\ln\chi -{\left(1-\chi \right)}^{2}\ln\left(1-\chi \right).$

Similarly, the semi-entropy of background asset

(19) $$s_{e}\left(r_{b}\right)=\left\{\begin{array}{cc} \left(\xi^{b}-\xi^{a}\right)\rho -\zeta \left(\rho_{b}\right), & \mathrm{if}\;E\left(r_{b}\right)\leq \xi^{b},\;\\ \frac{\xi^{b}-\xi^{a}}{2}+\frac{(\xi^{a}+\xi^{c}+\xi^{d}-3\xi^{b})\ln2}{4}, & \mathrm{if}\;\xi^{b}<E\left(r_{b}\right)\leq \xi^{c},\\ \frac{\xi^{b}-\xi^{a}}{2}+\left(\xi^{c}-\xi^{b}\right)\ln2+\zeta \left(\tau_{b}\right), & \mathrm{otherwise}, \end{array}\right.$$

where $\rho_{b}=\left(\xi^{b}+\xi^{c}+\xi^{d}-3\xi^{a}\right)/8\left(\xi^{b}-\xi^{a}\right)$, $\tau_{b}=\left(3\xi^{d}-\xi^{a}-\xi^{b}-\xi^{c}\right)/8\left(\xi^{d}-\xi^{c}\right)$, and $\zeta \left(\chi \right)=\chi^{2}\ln\chi -{\left(1-\chi \right)}^{2}\ln\left(1-\chi \right)$.

### 3.3. Constraints

- Liquidity
  In the process of making a portfolio decision, one of the key elements that should be considered is liquidity for investors. It measures the degree of probability that investors will convert an asset into income. Investors prefer assets with higher liquidity because their returns tend to rise over time. Generally, liquidity is measured by the turnover rate of assets. Because turnover rates cannot be precisely predicted, we suppose that the turnover rates of risky assets are fuzzy variables characterized by trapezoidal numbers. On account of the former discussion, by Equation (5), the constraint of the portfolio liquidity is expressed as
  (20) $$E\left(\sum_{i=1}^{n}x_{it}L_{it}\right)=\sum_{i=1}^{n}x_{it}\left(\frac{\beta_{it}^{a}+\beta_{it}^{b}+\beta_{it}^{c}+\beta_{it}^{d}}{4}\right)\geq L_{t},t=1,\ldots T.$$
- The desired number of risky assets that are selected into the portfolio during the t-th investment period is expressed as
  (21) $$\sum_{i=1}^{n}m_{it}=Z,i=1,2,\ldots n,t=1,2,\ldots T.$$
- The risk-free asset constrained in each period is
  (22) $$\sum_{i=1}^{n}x_{it}<1,i=1,2,\ldots n,t=1,2,\ldots T.$$
- The lower and upper limits that can be assigned to the i-th risky asset during the t-th investment period are given as
  (23) $$lb_{it}\leq x_{it}\leq ub_{it},i=1,2,\ldots n,t=1,2,\ldots T.$$
- Whether the i-th risky asset is selected into the portfolio during the t-th investment period is shown as
  (24) $$m_{it}\in \left\{0,1\right\},i=1,2,\ldots n,t=1,2,\ldots T.$$
- No short selling of assets during any investment period
  (25) $$x_{it}\geq 0,i=1,2,\ldots n,t=1,2,\ldots T.$$

### 3.4. The Proposed Model

Over the entire investment horizons, investor intends to obtain the greatest final wealth and minimize the risk at the same time to find a first-rank invest strategy. Then, we supply the multi-objective model for multi-period portfolio selection problems in the following:

(26) $$\begin{array}{cc} Max & \;W_{1}\prod_{t=1}^{T}\left(1+\sum_{i=1}^{n}\left(\frac{\alpha_{it}^{a}+\alpha_{it}^{b}+\alpha_{it}^{c}+\alpha_{it}^{d}}{4}\right)x_{it}-\sum_{i=1}^{n}f_{it}\left|x_{it}-x_{it-1}\right|\right.\\ \; & \left.+\frac{\xi_{a}+\xi_{b}+\xi_{c}+\xi_{d}}{4}+\left(1-\sum_{i=1}^{n}x_{it}\right)r_{f}\right),\\ Min & \;\sum_{t=1}^{T}\left[s_{e}\left(\sum_{i=1}^{n}x_{it}r_{it}\right)+s_{e}\left(r_{b}\right)\right]\\ \; & \mathrm{subject}\;\mathrm{to}\\ \; & \mathrm{Constraints}\;\left(20\right)-\left(25\right) \end{array}$$

$s_{e}\left(r_{it}\right)$ and $s_{e}\left(r_{b}\right)$ in the proposed model are defined by Equations (18) and (19), respectively.

## 4. The Proposed Hybrid Algorithm

### 4.1. Standard Dragonfly Algorithm (DA)

The static and dynamic swarming behaviors of dragonflies inspire the DA algorithm. These two behaviors represent the exploration phase and the exploitation phase, which are two major phases of the meta-heuristic algorithm. Five diverse operators determine the movement of swarm dragonflies:

- Separation
  For the individual *i*, its separation is calculated as $S_{i}=-\sum_{k=1}^{N}(P-Pn_{k})$. $Pn_{k}$ denotes the k-th adjacent individual’s position. *P* denotes the current individual’s position. *N* is the number of neighboring individuals.   
- Alignment
  For the individual *i*, its alignment is given as $A_{i}=\sum_{k=1}^{N}V_{k}/N,$ where $V_{k}$ is the velocity of the neighboring individual *k*.   
- Cohesion
  For the individual *i*, the cohesion is calculated as $C_{i}=\sum_{k=1}^{N}Pn_{k}/N-P.$   
- Attraction and Distraction
  For the individual *i*, the index for an individual being attracted by a food source is evaluated as $F_{i}=P^{+}-P$, where $P^{+}$ is the food source’s position. In addition, the index for an individual fleeing an enemy is calculated as $E_{i}=P^{-}+P$, where $P^{-}$ is the enemy’s position.

In order to find some new individuals in the search space, two vectors are employed. The step vector ΔP is used to update the locations of individuals, and the position vector *P* is introduced for simulating movements of the individuals. The movement directions of the individuals are given by the ΔP. If an individual has at least one neighbor, then ΔP is evaluated as

(27) $$\Delta P_{t+1}=\left(sS_{i}+aA_{i}+cC_{i}+fF_{i}+eE_{i}+\omega \Delta P_{t}\right).$$

In Equation (27), the separation weight is indicated by *s*, the alignment weight is shown by *a*, the cohesion weight is represented by *c*, and the food element and the enemy element are denoted as *f* and *e*, respectively. Furthermore, *t* is the iteration counter. According to ΔP in Equation (27), *P* is given as

(28) $$P_{t+1}=P_{t}+\Delta P_{t+1}.$$

If an individual has no neighbors, the Lévy Flight equation will be applied to update *P*. This equation can improve the randomness, global search capacity and chaotic behavior of individuals. *P* is calculated as

(29) $$P_{t+1}=P_{t}+L\overset{´}{e}vy\left(d\right)P_{t}.$$

In Equation (29), the equation of Lévy flight is

(30) $$L\overset{´}{e}vy\left(\chi \right)=0.01\times \frac{\eta_{1}\times \gamma }{|\eta_{2}{|}^{\frac{1}{\vartheta }}}.$$

In Equation (30), $\eta_{1}$ and $\eta_{2}$ are two random numbers taking values in [0,1], and ϑ is a constant, γ is calculated as

(31) $$\gamma ={\left(\frac{\Gamma \left(1+\vartheta \right)\times \sin\left(\frac{\pi \vartheta }{2}\right)}{\Gamma \left(\frac{1+\vartheta }{2}\right)\times \vartheta \times 2^{\left(\frac{\vartheta -1}{2}\right)}}\right)}^{\frac{1}{\vartheta }},$$

where Γ(χ)=(χ+1)!.

### 4.2. The Hybrid DA-GA for the Proposed Model

A good metaheuristic algorithm should better balance exploration and exploitation processes. The exploration process is used to investigate the new search space to find great global optima, while the exploitation process is used to focus on the search of local areas. Excessive exploitation results in premature convergence, while overmuch exploration leads to slow convergence. DA has advantages in exploring the global search space by using the food source and enemy source. However, the use of Lévy Flight results in a large movement that leads to local convergence and pushes the algorithm apart from the global optimum  [56]. In addition, NSGA II, developed by Deb et al.  [63], is a well-known meta-heuristic approach for solving multi-objective optimization problems. It has an improved mechanism that depends on the non-domination rank and the crowding distance and conducts constraints by using an adapted explanation of dominance instead of the penalty functions. Thus, NSGA II has a good ability to attain diverse and uniformly distributed Pareto solutions. In this paper, for the sake of solving the proposed model efficiently, a novel hybrid algorithm named HDA-GA is developed by combining the strengths of DA and NSGA II.

#### 4.2.1. Parameter Optimization

In the static swarm of DA, the probability of alignments is low, while the probability of cohesion is high. In order to enhance the information exchange of the dragonflies from global exploration to local exploitation, dragonflies are assigned with higher alignment weights and lower cohesion weights when the global space is explored and designed on the contrary when the local area is exploited. Therefore, the exponential function is introduced to adjust the swarming elements *a* and *c*. The factors *a* and *c* are given as follows:

(32) $$\begin{array}{cc} \; & a=e^{-h}, \end{array}$$

(33) $$\begin{array}{cc} \; & c=e^{h}, \end{array}$$

where *h* is adaptively decreased as the iteration increases.

Moreover, in order to enhance the randomness, the standard DA selected the positions of food source $P^{+}$ and enemy $P^{-}$ by using a roulette-wheel mechanism. However, in the global search space, it may lead to poor exploration ability. Inspired by the ideas in [51], we propose a new method for choosing food sources and enemies. $P_{gbest}$ and $P_{gworst}$ are defined as the best and the worst solutions in each iteration. The selections of $P^{+}$ and $P^{-}$ are given as follows:

(34) $$\begin{array}{cc} \; & P^{+}=P_{gbest}, \end{array}$$

(35) $$\begin{array}{cc} \; & P^{-}=P_{gworst}. \end{array}$$

#### 4.2.2. Constraints Handling

Note that the standard DA only considered the non-constrained situation. However, there exist constraints in the proposed model that cannot be ignored. In this paper, to handle the constraints, we employ the constrained domination approach proposed by Deb et al. [63].

If any of the conditions below is true, a solution $S_{k}$ is constrained-dominated by another solution $S_{j}$. (1) Both solutions are feasible, and solution $S_{k}$ is dominated by solution $S_{j}$; (2) The feasible solution is $S_{j}$, but the infeasible one is $S_{k}$; (3) Both are infeasible, but comparing the constrained violations of these two solutions, the violation solution $S_{j}$ has is smaller.

For the t−th inequality constraint $g_{t}\left(s\right)\leq 0$ and equality constraint $h_{t}\left(s\right)=0$, the constrained violation is estimated as

(36) $$CV_{t}=\left\{\begin{array}{cc} \; & max\{0,g_{t}\left(s\right)\},\;t=1,2,\ldots G,\\ \; & max\{h_{t}\left(s\right)-\iota ,0\},\;t=G+1,\ldots G+H, \end{array}\right.$$

where ι is a tolerance coefficient that violates the equality constraints. After the normalization of $cv_{t}$, the constrained violation of solution $S_{j}$ is given as

(37) $$CV_{j}=\sum_{t=1}^{G+H}CV_{t}.$$

For the purpose of drifting the solutions towards the Pareto front and making the Pareto-optimal set as diverse as possible, a joint strategy combining the constrained non-dominated sorting and crowding distance assignment is implemented. In the strategy, how close a solution is to its neighbors is measured by crowding distance $distance_{k}$. Diversity improves with larger $distance_{k}$. In the proposed algorithm, the crowding distance $distance_{k}$ measure introduced by Deb et al.  [63] is employed and calculated as follows:

(38) $$\begin{array}{c} distance_{k}=\frac{F_{1}\left(k+1\right)-F_{1}\left(k-1\right)}{F_{1max}-F_{1min}}+\frac{F_{2}\left(k+1\right)-F_{2}\left(k-1\right)}{F_{2max}-F_{2min}}. \end{array}$$

In Equation (38), the maximum and minimum of the first objective function is shown as $F_{1max}$ and $F_{1min}$, respectively. Similarly, the maximum and minimum of the second objective function are illustrated as $F_{2max}$ and $F_{2min}$, respectively. The constrained non-dominated sorting pseudo-code is summarized as Algorithm 1.

**Algorithm 1** Constrained non-dominated sorting.
1:Classify feasible and infeasible groups in the population by Equation (37)2:**For**p=1 to feasible_population do3:  Calculate $S_{p}$, a set of solutions that the p−th individual dominates4:  Calculate $n_{p}$, the number of individuals that dominate the p−th individual5:
**End for**
6:Create first front whose $n_{p}=0$7:**While** ($n_{p}>0$)8:  Create subsequent fronts by traversing $S_{p}$9:  Crowding distance assignment by Equation (38)10:
**End While**
11:**For**q=1 to infeasible_population do12:  Sort infeasible individual by Equations (36) and (37)13:
**End for**
14:Combine the feasible and infeasible solutions


#### 4.2.3. External Archive

An external archive is widely used to solve multi-objective problems and to maintain the Pareto optimal solutions during optimization. The standard MODA applies an archive to retain the best elite solutions and updates the archive with respect to the non-dominated sorting. However, the updating progress deletes the infeasible solutions directly. It did not consider the constrained situation either. Based on constrained dominate rules and crowding distance, an external archive is used to improve the speed of convergence and retain the diversity of the solution set. The archive is divided into two subsets, $Archive_{1}$ and $Archive_{2}$. $Archive_{1}$ saves solutions obtained by DA, while $Archive_{2}$ saves solutions solved by NSGA II. Finally, $Archive_{1}$ and $Archive_{2}$ make up a new set New_Archive for the next generation. Initially, this archive is empty. As the iteration goes by, feasible and infeasible solutions enter the archive, and the size of the archive may be huge. If the archive is full, one or more than one solution may be deleted. The progress of this method is summarized as pseudo-code shown in Algorithm 2.

Through the above discussions, Algorithm 3 describes the proposed hybrid algorithm. In the hybrid algorithm, both DA and NSGA II start with the same initial population. The external archive is divided into two parts, where one retains feasible solutions and the other saves infeasible solutions during each iteration. Each of the two parts is evolved by a respective algorithm and then recombined in the updating archive process.

**Algorithm 2** Update archive.
1:Classify the population by Equation (37)2:Divide the archive to $Archive_{1}$ whose $CV_{t}=0$ and $Archive_{2}$ Whose $CV_{t}\neq 0$3:**While** ($N_{Archive_{1}}>0$)4:  estimate the rank of each solution according to the Equation (37)5:  Constrained non-dominated sorting by Algorithm 16:  Calculate the crowding distance by Equation (38)7:
**End While**
8:**While** ($N_{Archive_{2}}>0$)  Sort by Equation (37)   Set the distance to inf9:
**End While**



**Algorithm 3** The pseudo-codes of the HDA-GA.
1:Define the max_iter, ArchiveMaxSize, ub, lb and *r*2:Initialize $X_{i}$ by $X_{i}=random*\left(ub-lb\right)+lb$ and $\Delta X_{i}$ by $\Delta X_{i}=random*\left(ub-lb\right)+lb$3:Calculate the initialized objective function values4:Initialized constrained non-dominated sorting by Algorithm 15:**While** (t≤max_iter)6:  Update neighboring radius and the factors w,s,a,c,fe7:  Calculate the objective function values8:  Update The Archive with respect to Algorithm 29:  Select the Food source and Enemy from Archive110:  **If** Archive≤ArchiveMaxSize11:   Select individuals from the particular front based on crowding distance by Equation (38)12:  **end if**13:  **For** i=1 to Archive1 do14:   Find their neighbors with respect to the Euclidean distance15:   Calculate S,A,C,FandE16:   **If** an individual has one neighbor at least17:    Update $\Delta X_{t}$ by Equation (27) and $X_{t+1}$ by Equation (28)18:   **end if**19:   **If** an individual has no neighbor20:    Update $X_{t+1}$ by Equation (29)21:   **end if**22:  **end for**23:  **For** j=1 to Archive224:   Selected()25:   Crossover()26:   Mutation()27:  **end for**28:
**End While**



## 5. Numerical Experiments

For the sake of verifying the usefulness of the proposed methods, numerical empirical examples introduced by Mehlawat [42] are presented. The fuzzy return rates of the 10 risky assets in each period are presented in Table 1, and Table 2 shows the fuzzy turnover rates of these 10 risky assets. The background asset returns are given by experts’ estimations.

In this empirical study, we hypothetically set the initial wealth as $W_{1}=1$, the lower and upper bounds are set as $u_{it}=0.1$ and $l_{it}=0.5$, respectively, the unit transaction cost is $f_{t}=0.003$, and the desired number of risky assets chosen for the portfolio during the t-th investment period is $Z_{t}=5$. In addition, we assume that n=10 and T=3. The fuzzy variable $r_{b}=\left(0.080,0.090,0.109,0.121\right)$ is the return rate on a background asset, the return rate on risk-free assets is $r_{f}=0.01$, and the accepted minimum expected liquidities during each investment interval are designed as $L_{1}=0.0045$, $L_{2}=0.0035$, and $L_{3}=0.0025$.

### 5.1. Parameter Settings

Six algorithms, HDA-GA, NSGA II [63], the multi-objective dragonfly algorithm (MODA) [51], the multi-objective particle swarm algorithm (MOPSO) [50], the multi-objective salp swarm algorithm (MOSSA) [47], and the multi-objective artificial bee algorithm (MOABC) [48], are compared in these experiments. The parameters of each algorithm are set as follows:

HDA-GA: population_size=100, max_iter=400, the probability of individual mutation $p_{m}=1/n$, the crossover distribution exponent etac=20, and the mutation distribution exponent etam=100.

The parameters in NSGA II and MODA are equal to those in HDA-GA.

MOPSO: The modulus of personal learning c1 is 1, the modulus of global learning c2 is 2, and the initial weight *w* is 0.5.

MOSSA: The initial range *r* is 0.2, and the initial max velocity $V_{max}$ is 0.04.

MOABC: The food_Number is 200, and the limit is 50.

In addition, each algorithm independently runs 30 times, and the average results are obtained after running.

### 5.2. Performance Measure Metrics

Five performance metrics, GD, Spacing, Diversity, CM and MPFE, are selected to compare the performances of the algorithms.

Generation Distance (GD): This convergence metric is employed to compute the distance between the approximated Pareto frontier and the true Pareto frontier. It is calculated as [63]

(39) $$GD=\frac{\sqrt{\sum_{m=1}^{N}d_{m}^{2}}}{N},$$

where *N* is the number of the obtained solutions, and $d_{m}$ is the minimum Euclidean distance between each of the obtained solutions and the true Pareto frontier. A smaller value of GD means that the obtained Pareto frontier is closer to the true Pareto frontier.

Spacing: This diversity metric is applied to measure the propagate of the obtained values. It is evaluated as [64]

(40) $$Spacing=\sqrt{\frac{1}{N-1}\sum_{k=1}^{N}{\left(d_{ave}-d_{k}\right)}^{2}},$$

where $d_{k}$ is the minimum distance between the k−th solution and its adjacent solutions, $d_{k}=\min_{i}\left(\sum_{s=1}^{N}|F_{s}^{i}-F_{s}^{j}\right|)$, and $d_{ave}$ is the average distance of $d_{k}$. A smaller value of Spacing indicates that the obtained solutions are in a better distribution.

Diversity: This diversity metric measures the spread and distribution of the obtained solutions. It is given as [63]

(41) $$Diversity=\frac{d_{e}+d_{b}+\sum_{k=1}^{N-1}\left|d_{k}-d_{ave}\right|}{d_{e}+d_{b}+\left(N-1\right)d_{ave}},$$

where $d_{e}$ and $d_{b}$ are the distance between the boundary of the obtained solutions and the extreme values of the true Pareto frontier. A smaller value of Diversity means a better distribution and spread of obtained solutions.

Convergence Metric (CM): This convergence metric measures the extent of convergence to the true Pareto frontier. It is computed as [63]

(42) $$CM=\frac{\sum_{m=1}^{N}d_{m}}{N},$$

where $d_{m}$ is the Euclidean distance between the solution obtained with the algorithm and the nearest solution on the Pareto frontier. The smaller the value of this metric is, the better the convergence toward the true Pareto frontier.

Maximum Pareto front error (MPFE): This convergence-diversity metric is employed to measure the quality of the obtained solutions in terms of diversity and convergence on a single scale. It is expressed as [64]

(43) $$MPFE=\max_{P}\sqrt{\min_{s}\sum_{q=1}^{Q}{\left(F_{q}^{s}-F_{q}^{p}\right)}^{2}},$$

where *Q* is the number of objective functions and *P* is the number of the Pareto solutions. MPFE aims to find the maximum minimum distance between each solution obtained with the algorithm and the corresponding nearest solution on the Pareto frontier. The convergence and the diversity of the algorithm improve with smaller values of this metric.

### 5.3. Experimental Results Based on the Zdt Functions

In this section, we select four ZDT functions as benchmarks and present a comparison of these functions to verify the validity of the proposed HDA-GA. The details of the four ZDT functions are in Appendix A. Table 3 and Table 4 show the best (Best), mean (Mean) and standard deviation (SD) of the five performance metrics. The bold fonts indicate better results. It can be easily observed that the proposed HDA-GA is superior to the other five algorithms within the five performance metrics.

ZDT1 is a relatively easier problem than the other three ZDT problems. From Table 3, MOPSO and MOSSA have better SD than HDA-GA. However, HDA-GA has the smallest Mean of the five metrics among the six algorithms, which means that HDA-GA converges to the Pareto frontier with the best distribution, spread, and diversity.

Five disjoint curves make up the Pareto front of ZDT3. With respect to GD and Diversity, although MOPSO can obtain the Best, HDA-GA performs better between Mean and SD. In addition, HDA-GA has the smallest Best and Mean of two metrics, CM and MPFE. Moreover, HDA-GA owns a better Spacing than others, which means solutions produced by HDA-GA have a better distribution than others.

ZDT6 is another difficult problem for many multi-objective optimization algorithms to achieve a set of solutions with good convergence and diversity. From Table 4, for GD and CM, although MOPSO has smaller SD, HDA-GA performs better in Best and Mean than the others. For diversity metrics Spacing and Diversity, solutions produced by HDA-GA spread out better over the Pareto frontier with a better distribution. The results of MPFE demonstrate a superior convergence and diversity ability of HDA-GA.

For ZDT2, although MOPSO and MOSSA perform more stably than HDA-GA with respect to GD and Spacing, HDA-GA has the smallest Mean, Best and SD of Diversity, CM and MPFE, which indicates that HDA-GA finds a better distribution and spread with a smaller convergence metric than others.

Based on the above discussion, HDA-GA has a superior convergence and diversity ability with a better distribution and spread. It indicates that HDA-GA outperforms the other algorithms in most of the performance metrics.

### 5.4. Experimental Results Based on the Proposed Model

This section presents three cases with different cardinality constraints. For the proposed model, the minimum (Min), maximum (Max), mean (Mean), standard deviation (SD) and range (Range) of the results found by six different algorithms are revealed in Table 5. The bold fonts indicate better results. Given the comparisons among the six algorithms, HDA-GA can own the smallest mean value in all the cases. In addition, according to the comparison of min and max index, we can see that HDA-GA can acquire a set of non-dominated solutions with better distribution. Finally, the comparison of Range index illustrates that HDA-GA can search space reliably and extensively. Although the MODA is more stable than the HDA-GA in terms of SD index, it is easier for MODA to fall into local optimization. These results indicated that HDA-GA performs better than the other algorithms.

Moreover, for a fair comparison of the performances among the algorithms, GD, Spacing, Diversity, CM, and MPFE are employed as the performance measurement metrics. Table 6 presents some results in terms of the five metrics above. For GD and CM, the index values indicate results obtained by the proposed HDA-GA are closer to the Pareto front than the other algorithms in the three cases. Meanwhile, for Spacing and Diversity, HDA-GA performs better than the other algorithms, which means that it finds a better spread and distribution metric than others. Moreover, for MPFE, HDA-GA has a superior convergence and diversity ability.

**Figure 1:**
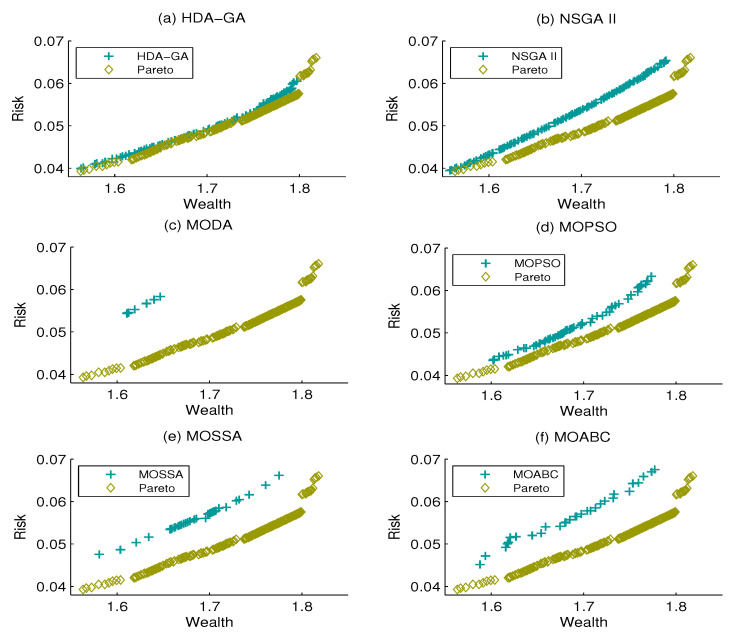
The approximate Pareto front and six algorithm efficient front when Z = 3.

**Figure 2:**
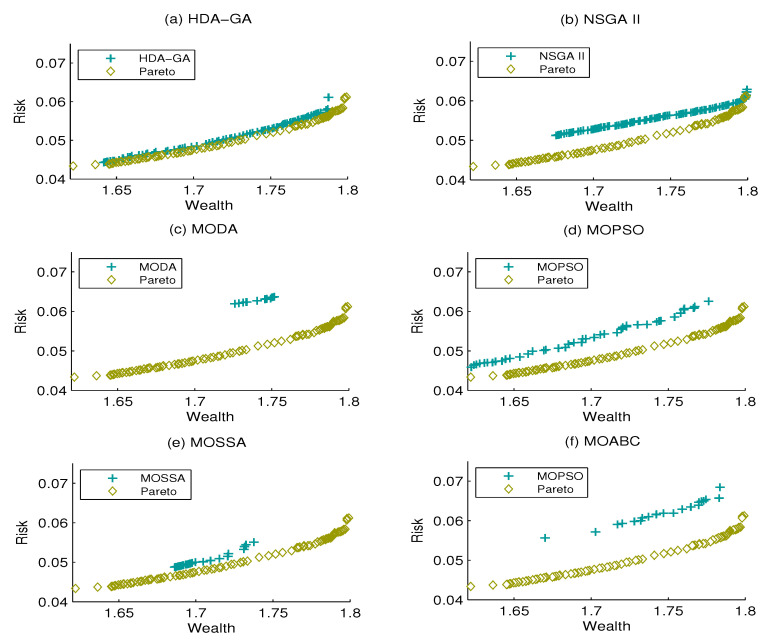
The approximate Pareto front and six algorithm efficient front when Z = 5.

**Figure 3:**
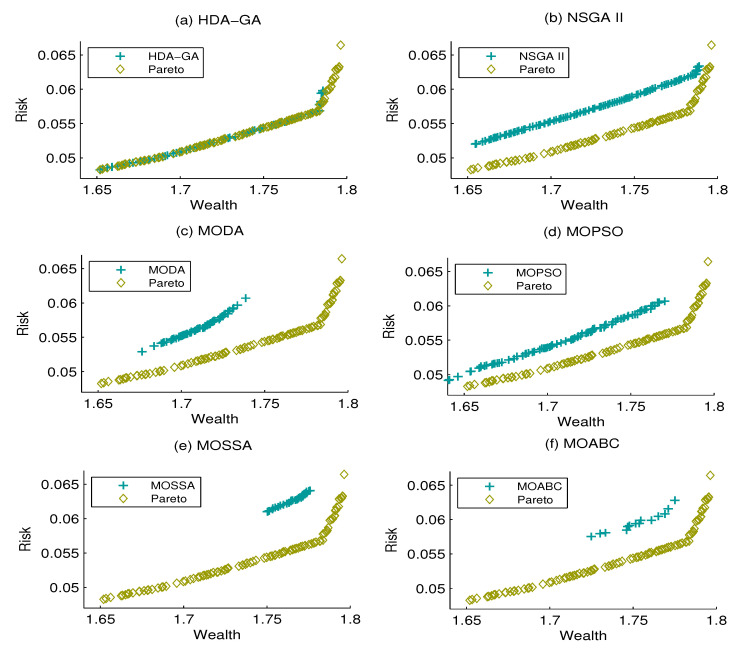
The approximate Pareto front and six algorithm efficient front when Z = 7.

Figure 1, Figure 2 and Figure 3 display the Pareto front and the efficient frontiers of the six algorithms under the three cases above. It can be seen that the proposed HDA-GA can obtain a set of non-dominated solutions that approach the Pareto front properly. Moreover, we can see that the proposed HDA-GA performs better with accurate convergence, preferable coverage, and better diversity.

### 5.5. Experimental Results with and without Background Risk

We present four cases to analyze the impact of background risk in the proposed model. Case 1: Without background risk asset (BR); Case 2: With background risk asset BR1 whose fuzzy return is $r_{b_{1}}=\left(0,024,0.027,0.0327,0.0363\right)$; Case 3: With background asset BR2 whose fuzzy return is $r_{b_{2}}=\left(0,040,0.045,0.0545,0.0605\right)$; Case 4: With background asset BR3 whose fuzzy return is $r_{b_{3}}=\left(0,080,0.090,0.109,0.121\right)$. The experimental results indicate that the background risk has a significant impact on the portfolio selection.

From Table 7, it can be observed that cases considering background risk have higher returns and risk than that without background risk. Ignoring background risk will cause the underestimation of risk and the reduction of return in the actual investment.

**Figure 4:**
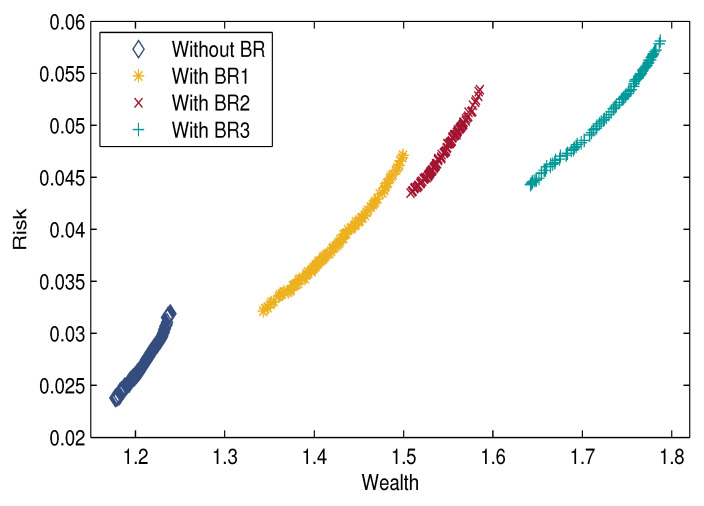
The Pareto frontier of the mean-semi entropy model with and without background risk.

In addition, Figure 4 shows the Pareto frontiers of the above four cases. The shapes of the Pareto frontiers are approximately the same, and the Pareto frontier moves right as the background risk is concerned. It can be observed that there is a positive correlation between the background asset return and portfolio return. When the risk is the same, a portfolio with background risk can obtain a higher return than that without background risk. It indicates that considering background risk avoids the reduction of return in the actual investment and the ignorance of the potential income in the actual investment. Moreover, the risk of background assets is positively correlated with portfolio risk. When the return is the same, a portfolio with background risk is riskier than one without background risk. Considering background risk can prevent investors from underestimating the investment risk and ignoring the potential risk.

## 6. Conclusions

In the real world, investors usually need to optimize the portfolio strategies from time to time. In this paper, we proposed a mean-semi-entropy model based on the credibility theory by taking buy-in thresholds, cardinality, liquidity, and transaction costs into account. In particular, background risk is also considered in the proposed model. To solve the proposed multi-objective model, a hybrid algorithm, HDA-GA, combining the advantages of dragonfly algorithm (DA) and non-dominated sorting genetic algorithm II (NSGA II), is developed. Finally, we conducted a series of experiments to demonstrate the effectiveness of the proposed model and the hybrid algorithm. The numerical results showed that (1) the proposed algorithm HDA-GA is superior to the other five algorithms, namely, NSGA II, MODA, MOPSO, MOSSA, and MOABC, with accurate convergence, preferable coverage, and better diversity; (2) the mean-semi-entropy model can lead to more distributive investments; and (3) considering background risk will prevent investors from the underestimation of risk in the actual investment.

Future research directions include but are not limited to the following: (1) considering a more general transaction cost structure as in Beraldi et al. [12]; (2) extending the proposed model by adding other constraints of real markets such as minimum transaction lots, skewness, and class constraints; and (3) applying other metaheuristic algorithms such as the estimation of distribution algorithm (EDA), the krill herd (KH) algorithm, and bacterial foraging optimization (BFO) for solving the proposed model.

## Figures and Tables

**Table 1:** The fuzzy returns of 10 risky assets at each period.

Asset	t=1	t=2	t=3
A1	(0.08026, 0.10069, 0.12130, 0.13173)	(0.10026, 0.12207, 0.13013, 0.15017)	(0.09026, 0.10691, 0.12513, 0.13452)
A2	(0.09829, 0.11543, 0.12143, 0.14589)	(0.06258, 0.08535, 0.10541, 0.15459)	(0.08829, 0.10525, 0.12520, 0.15259)
A3	(0.07615, 0.11306, 0.13807, 0.16765)	(0.09124, 0.11256, 0.13251, 0.14215)	(0.07159, 0.09031, 0.12945, 0.14255)
A4	(0.09381, 0.12810, 0.14143, 0.16572)	(0.09371, 0.11810, 0.12714, 0.13257)	(0.08381, 0.10810, 0.11271, 0.13157)
A5	(0.08967, 0.10913, 0.12837, 0.14783)	(0.10260, 0.11569, 0.12564, 0.14625)	(0.09130, 0.11234, 0.12645, 0.15978)
A6	(0.06357, 0.09286, 0.11786, 0.15772)	(0.07357, 0.09265, 0.11246, 0.13976)	(0.09584, 0.10563, 0.12622, 0.15561)
A7	(0.04961, 0.08562, 0.10804, 0.13464)	(0.09961, 0.10562, 0.12880, 0.14841)	(0.09961, 0.10562, 0.11380, 0.12541)
A8	(0.08464, 0.11570, 0.12319, 0.16425)	(0.09464, 0.11206, 0.12232, 0.14425)	(0.05464, 0.07014, 0.09319, 0.10643)
A9	(0.05946, 0.08855, 0.10729, 0.12638)	(0.08240, 0.10974, 0.11322, 0.14494)	(0.07240, 0.08597, 0.12202, 0.14936)
A10	(0.05311, 0.09298, 0.11933, 0.13920)	(0.09036, 0.10410, 0.11179, 0.12239)	(0.06311, 0.08298, 0.10259, 0.12892)

**Table 2:** The fuzzy turnover rates of 10 risky assets at each period.

Asset	t=1	t=2	t=3
A1	(0.00106, 0.00282, 0.00528, 0.00704)	(0.00101, 0.00276, 0.00517, 0.00690)	(0.00079, 0.00217, 0.00406, 0.00542)
A2	(0.00031, 0.00083, 0.00156, 0.00208)	(0.00028, 0.00074, 0.00139, 0.00185)	(0.00033, 0.00087, 0.00164, 0.00218)
A3	(0.00365, 0.00973, 0.01825, 0.02433)	(0.00310, 0.00827, 0.01551, 0.02068)	(0.00383, 0.01071, 0.02007, 0.02677)
A4	(0.00143, 0.00382, 0.00717, 0.00956)	(0.00122, 0.00337, 0.00631, 0.00841)	(0.00136, 0.00352, 0.00653, 0.00870)
A5	(0.00114, 0.00305, 0.00572, 0.00763)	(0.00143, 0.00382, 0.00658, 0.00954)	(0.00116, 0.00308, 0.00578, 0.00771)
A6	(0.00189, 0.00505, 0.00947, 0.01262)	(0.00218, 0.00581, 0.01089, 0.01451)	(0.00199, 0.00530, 0.00994, 0.01325)
A7	(0.00130, 0.00348, 0.00652, 0.00869)	(0.00102, 0.00285, 0.00535, 0.00678)	(0.00137, 0.00365, 0.00685, 0.00913)
A8	(0.00413, 0.01102, 0.02067, 0.02756)	(0.00356, 0.00948, 0.01819, 0.02425)	(0.00380, 0.01014, 0.01943, 0.01943)
A9	(0.00100, 0.00267, 0.00501, 0.00668)	(0.00101, 0.00272, 0.00511, 0.00688)	(0.00095, 0.00246, 0.00461, 0.00634)
A10	(0.00151, 0.00403, 0.00755, 0.01007)	(0.00159, 0.00419, 0.00808, 0.01078)	(0.00141, 0.00367, 0.00703, 0.00927)

**Table 3:** Performance measure metrics of six algorithms on ZDT1 and ZDT2.

			HAD-GA	NSGA II	MODA	MOPSO	MOSSA	MOABC
ZDT1	GD	Best	**0.004095**	0.012602	0.023130	0.005491	0.008020	0.008363
		Mean	**0.010285**	0.031497	0.011441	0.030793	0.011919	0.015718
		SD	0.005617	0.015824	0.007683	0.015226	**0.003069**	0.009775
	Spacing	Best	0.005900	0.008365	0.012568	**0.004856**	0.007206	0.011102
		Mean	**0.010355**	0.071218	0.020328	0.052385	0.013950	0.016263
		SD	**0.003716**	0.051334	0.072608	0.008272	0.003925	0.004636
	Diversity	Best	**0.689252**	0.767096	1.056672	0.928113	0.998926	0.710941
		Mean	**0.777430**	1.191056	0.952947	0.877077	1.061126	0.808524
		SD	0.047591	0.095529	0.109909	**0.016630**	0.027691	0.085882
	CM	Best	**0.029047**	0.044361	0.153425	0.044849	0.068340	0.066786
		Mean	**0.085282**	0.122808	0.248206	0.099676	0.097692	0.096000
		SD	0.052282	0.066176	0.048990	0.138730	**0.023838**	0.025184
	MPFE	Best	**0.00874**	0.009992	0.011139	0.008925	0.009385	0.00959
		Mean	**0.013108**	0.020181	0.133448	0.015529	0.015717	0.031373
		SD	0.194764	0.013881	0.889816	**0.003105**	0.009057	0.04551
ZDT2	GD	Best	**0.005093**	0.020029	0.024264	0.005898	0.006069	0.006479
		Mean	**0.006309**	0.051167	0.043403	0.007023	0.020066	0.023395
		SD	0.002625	0.020365	0.018287	**0.001099**	0.017294	0.129870
	Spacing	Best	**0.006159**	0.007313	0.014746	0.010294	0.009619	0.919671
		Mean	**0.008166**	0.054016	0.117920	0.017602	0.013638	0.064095
		SD	0.003014	0.077159	0.206688	0.006310	**0.002957**	0.078074
	Diversity	Best	**0.743251**	0.756327	1.021222	0.887392	1.016217	0.784003
		Mean	**0.755997**	0.923211	1.186858	0.947667	1.054720	0.919671
		SD	**0.010433**	0.122369	0.115658	0.034393	0.029056	0.113666
	CM	Best	**0.040808**	0.120144	0.186254	0.051457	0.042942	0.065679
		Mean	**0.043401**	0.252616	0.331772	0.061296	0.103852	0.044393
		SD	**0.003348**	0.092178	0.124765	0.010139	0.007846	0.053198
	MPFE	Best	**0.002547**	0.019930	0.005916	0.008524	0.007652	0.010034
		Mean	**0.015176**	0.031135	0.382625	0.017301	0.050612	0.526517
		SD	**0.007083**	0.013584	0.507489	0.006949	0.075455	1.000795

**Table 4:** Performance measure metrics of six algorithms on ZDT3 and ZDT6.

			HAD-GA	NSGA II	MODA	MOPSO	MOSSA	MOABC
ZDT3	GD	Best	0.054976	0.053921	0.060290	**0.048783**	0.054016	0.076657
		Mean	**0.057206**	0.057536	0.066687	0.059919	0.057373	0.140799
		SD	**0.001461**	0.002703	0.004212	0.007767	0.001838	0.088669
	Spacing	Best	**0.003112**	0.003233	0.007077	0.010165	0.004314	0.013413
		Mean	**0.003780**	0.003812	0.014080	0.027349	0.009167	0.114245
		SD	**0.000384**	0.000575	0.007398	0.011083	0.002770	0.096158
	Diversity	Best	0.680960	0.701685	0.986596	**0.419654**	1.058633	0.697293
		Mean	**0.724077**	0.799906	1.066269	0.727388	1.092402	0.985891
		SD	**0.019516**	0.140285	0.051140	0.159316	0.030796	0.154812
	CM	Best	**0.040288**	0.439192	0.489842	0.086613	0.437459	0.099388
		Mean	**0.319770**	0.472352	0.545238	0.417218	0.462973	0.748504
		SD	0.184590	0.022893	0.035011	0.146361	**0.017012**	0.413275
	MPFE	Best	**0.037182**	0.346455	0.442562	0.040891	0.112903	0.046752
		Mean	**0.143945**	0.441165	0.462649	0.179891	0.402855	0.368856
		SD	0.018545	0.026356	**0.010757**	0.066136	0.096812	0.210216
ZDT6	GD	Best	**0.002196**	0.002822	0.033799	0.003517	0.016497	0.046591
		Mean	**0.004173**	0.012294	0.047519	0.004530	0.032995	0.097141
		SD	0.002653	0.013262	0.006311	**0.000839**	0.009279	0.035186
	Spacing	Best	0.005124	**0.003738**	0.003984	0.007407	0.005688	0.041192
		Mean	**0.005871**	0.005975	0.012857	0.008912	0.018166	0.234823
		SD	**0.000691**	0.000773	0.011254	0.000868	0.009442	0.100987
	Diversity	Best	**0.332990**	0.389267	0.943062	0.672322	0.962966	0.945702
		Mean	**0.415185**	0.529628	1.053706	0.791466	1.170424	1.293588
		SD	**0.044368**	0.145336	0.052485	0.052640	0.167927	0.231229
	CM	Best	**0.018328**	0.088036	0.178823	0.028255	0.135687	0.350734
		Mean	**0.037211**	0.173193	0.336264	0.037878	0.295232	0.652836
		SD	0.025601	0.119773	0.087734	**0.007565**	0.088986	0.291829
	MPFE	Best	**0.044172**	0.08062	0.382623	0.07309	0.160158	0.105027
		Mean	**0.073136**	0.160931	0.49514	0.075184	0.291002	0.274393
		SD	0.011518	0.120123	0.044012	**0.002094**	0.097323	0.114017

**Table 5:** Performance comparison among six different algorithms with different *Z*.

			HDA-GA	NSGAII	MODA	MOPSO	MOSSA	MOABC
Z = 3	Wealth	Min	**1.633495**	1.632514	1.550407	1.614602	1.530761	1.585684
		Max	**1.794225**	1.793599	1.621601	1.770328	1.771538	1.766484
		Mean	**1.720748**	1.720665	1.576514	1.692621	1.662877	1.687489
		SD	0.047306	0.048121	0.010112	**0.043021**	0.063723	0.050568
		Range	0.160729	0.161085	0.071193	0.155726	**0.240776**	0.180800
	Risk	Min	**0.044604**	0.046545	0.049435	0.049348	0.045243	0.047369
		Max	**0.062959**	0.063011	0.063454	0.064255	0.063346	0.06455
		Mean	**0.053731**	0.054231	0.054022	0.055233	0.054041	0.055383
		SD	0.004756	0.004895	**0.001089**	0.004442	0.006054	0.005441
		Range	**0.018355**	0.016467	0.014019	0.014907	0.018103	0.017181
Z = 5	Wealth	Min	1.678783	1.680510	**1.749434**	1.685986	1.695941	1.698884
		Max	**1.781419**	1.774321	1.756440	1.771944	1.757159	1.772371
		Mean	**1.735028**	1.731273	1.729111	1.728345	1.725990	1.733189
		SD	0.029946	0.027653	**0.001888**	0.026805	0.019354	0.021908
		Range	**0.102636**	0.093811	0.007006	0.085959	0.061218	0.073487
	Risk	Min	**0.050942**	0.053551	0.062524	0.053970	0.052848	0.056144
		Max	0.061726	0.063195	0.063153	0.062456	**0.061050**	0.063553
		Mean	**0.055967**	0.057751	0.062916	0.057906	0.056094	0.058972
		SD	0.003032	0.002664	**0.000175**	0.002585	0.002350	0.002017
		Range	**0.010784**	0.009643	0.000629	0.008486	0.008203	0.007409
Z = 7	Wealth	Min	1.660493	1.65951	1.671354	1.681038	**1.73473**	1.720436
		Max	**1.781933**	1.779803	1.717909	1.775401	1.779598	1.769565
		Mean	**1.73206**	1.72557	1.692663	1.726556	1.72578	1.73148
		SD	0.036646	0.036571	**0.011338**	0.028086	0.012798	0.014475
		Range	**0.121440**	0.120293	0.046555	0.094363	0.044868	0.049128
	Risk	Min	**0.052277**	0.052344	0.053272	0.053989	0.059038	0.058292
		Max	0.063028	0.062911	**0.058448**	0.063655	0.064246	0.063573
		Mean	**0.056600**	0.057057	0.056864	0.058096	0.061412	0.060949
		SD	0.003083	0.00295	**0.001212**	0.002772	0.001409	0.001512
		Range	**0.010750**	0.010567	0.005176	0.009666	0.005208	0.005281

**Table 6:** Performance metrics of the six algorithms on the mean-semi entropy model with different *Z*.

			HDA-GA	NSGAII	MODA	MOPSO	MOSSA	MOABC
Z = 3	GD	Best	**0.001138**	0.007173	0.008757	0.015083	0.009175	0.002060
		Mean	**0.006258**	0.009465	0.014247	0.020071	0.012466	0.007076
		SD	0.002285	**0.001191**	0.002916	0.002469	0.002299	0.003275
	Spacing	Best	0.000296	0.002106	0.000891	0.000205	0.001016	**0.000189**
		Mean	**0.000888**	0.005012	0.002801	0.005205	0.002556	0.001058
		SD	**0.000323**	0.002922	0.001977	0.003797	0.001802	0.001812
	Diversity	Best	**0.435084**	0.497652	0.639821	0.915870	0.997345	0.447954
		Mean	**0.613686**	0.653083	0.831236	1.104390	1.193895	0.691140
		SD	0.099787	0.117187	0.112371	0.136544	**0.084715**	0.155494
	CM	Best	**0.009877**	0.012099	0.065545	0.010396	0.010839	0.010649
		Mean	**0.017581**	0.038297	0.099294	0.034960	0.028905	0.018102
		SD	**0.003206**	0.019315	0.015591	0.012629	0.014536	0.005368
	MPFE	Best	**0.000863**	0.003766	0.012149	0.013759	0.023523	0.015990
		Mean	**0.007591**	0.008785	0.037146	0.068917	0.041119	0.028185
		SD	0.003098	**0.002667**	0.043248	0.048373	0.015846	0.008006
Z = 5	GD	Best	**0.001086**	0.002811	0.001848	0.002688	0.002466	0.001113
		Mean	**0.002115**	0.003583	0.004479	0.003567	0.002778	0.002491
		SD	0.000820	0.000523	0.003193	0.000380	**0.000199**	0.001141
	Spacing	Best	0.000205	0.001218	0.000238	0.000222	**0.000110**	0.000238
		Mean	**0.000259**	0.003500	0.000517	0.000344	0.000347	0.000300
		SD	**0.000045**	0.001966	0.000223	0.000539	0.011592	**0.000052**
	Diversity	Best	**0.459979**	0.525712	0.828962	0.999147	1.007670	0.557988
		Mean	**0.664117**	0.714527	0.960198	1.022843	1.029092	0.778562
		SD	0.089468	0.146712	0.061647	0.041030	**0.011592**	0.129619
	CM	Best	**0.015200**	0.019372	0.035838	0.018379	0.025984	0.018212
		Mean	**0.034833**	0.035417	0.057672	0.062308	0.035036	0.047096
		SD	0.011454	0.011889	**0.008579**	0.025695	0.010552	0.014139
	MPFE	Best	0.003708	0.004325	0.004665	**0.002945**	0.005110	0.004495
		Mean	**0.008469**	0.013077	0.010545	0.017373	0.009836	0.024503
		SD	**0.003230**	0.004618	0.004218	0.014576	0.003471	0.003540
Z = 7	GD	Best	**0.001366**	0.00162	0.005371	0.003709	0.001774	0.001997
		Mean	**0.002464**	0.002878	0.009859	0.007105	0.003042	0.002565
		SD	0.000655	0.000736	0.004902	0.002403	0.001323	**0.000356**
	Spacing	Best	0.00037	0.000543	0.000161	0.000263	**0.000153**	0.000662
		Mean	**0.000539**	0.000652	0.005097	0.001205	0.000549	0.004011
		SD	9.65E-05	**8.19E-05**	0.014545	0.001014	0.0006	0.002734
	Diversity	Best	**0.397332**	0.421402	0.820168	0.712305	0.820168	0.43828
		Mean	**0.569211**	0.656652	0.972133	0.919616	1.042666	0.777421
		SD	0.111023	0.082987	0.079158	0.178901	**0.058429**	0.178272
	CM	Best	**0.012427**	0.01507	0.046793	0.027399	0.017704	0.016771
		Mean	**0.023786**	0.027721	0.100074	0.048298	0.02411	0.024321
		SD	0.009023	0.007349	0.113323	0.012733	0.005012	**0.002925**
	MPFE	Best	**0.002981**	0.003287	0.00347	0.005616	0.00447	0.005449
		Mean	**0.006561**	0.006854	0.007169	0.009235	0.007457	0.007862
		SD	0.003014	0.002752	0.002962	0.005304	0.001864	**0.001628**

**Table 7:** Comparison of the proposed models with and without background assets.

		Without BR	With BR1	With BR2	With BR3
Wealth	Min	1.185022	1.380577	1.460292	1.678783
	Max	1.241051	1.493539	1.568064	1.781419
	Mean	1.215920	1.442530	1.520426	1.735028
	SD	0.015381	0.034563	0.031954	0.029946
Risk	Min	0.025120	0.039675	0.043203	0.050942
	Max	0.032879	0.051987	0.054544	0.061726
	Mean	0.028855	0.044879	0.048188	0.055967
	SD	0.002052	0.003497	0.003279	0.003032

## Abbreviations

The following abbreviations are used in this manuscript:

HDA-GA	Hybrid Dragonfly Algorithm-Genetic Algorithm
NSGA II	Non-Dominated Sorting Genetic Algorithm II
DA	Dragonfly algorithm
MODA	Multi-objective dragonfly algorithm
PSO	Particle swarm optimization
MOPSO	Multi-objective particle swarm optimization
SSA	Salp swarm algorithm
MOSSA	Multi-objective salp swarm algorithm
ABC	Artificial bee colony algorithm
MOABC	Multi-objective artificial bee colony algorithm
FA	Firefly algorithm
ED	Estimation of distribution algorithm
KH	Krill herd algorithm
BFO	Bacterial foraging optimization
VaR	Value at risk
CVaR	Conditional value at risk
LAD	Lower absolute deviation
BR	Background risk asset
GD	Generation distance
CM	Convergence Metric
MPFE	Maximum Pareto front error
SD	Standard deviation

## Appendix A. Multi-Objective Test Functions Utilized in This Paper

- ZDT1
  (A1) $$\begin{array}{cc} \; & Min\;f_{1}\left(x\right)=x_{1}\\ \; & Min\;f_{2}\left(x\right)=g\left(x\right)*\left(1-\sqrt{\frac{x_{1}}{g(x)}}\right)\\ \; & g\left(x\right)=1+\frac{9}{n-1}\sum_{i=2}^{n}x_{i},\\ \; & \mathrm{where}:\;0\leq x\leq 1,n=30. \end{array}$$
- ZDT2
  (A2) $$\begin{array}{cc} \; & Min\;f_{1}\left(x\right)=x_{1}\\ \; & Min\;f_{2}\left(x\right)=g\left(x\right)*\left[1-{\left(\frac{x_{1}}{g(x)}\right)}^{2}\right]\\ \; & g\left(x\right)=1+\frac{9}{n-1}\sum_{i=2}^{n}x_{i},\\ \; & \mathrm{where}:\;0\leq x\leq 1,n=30. \end{array}$$
- ZDT3
  (A3) $$\begin{array}{cc} \; & Min\;f_{1}\left(x\right)=x_{1}\\ \; & Min\;f_{2}\left(x\right)=g\left(x\right)*\left[1-\sqrt{\frac{x_{1}}{g(x)}}-\frac{x_{1}}{g(x)}*\sin\left(10\pi x_{i}\right)\right]\\ \; & g\left(x\right)=1+\frac{9}{n-1}\sum_{i=2}^{n}x_{i},\\ \; & \mathrm{where}:\;0\leq x\leq 1,n=30. \end{array}$$
- ZDT6
  (A4) $$\begin{array}{cc} \; & Min\;f_{1}\left(x\right)=1-exp\left(-4x_{1}\right)\sin^{6}\left(6\pi x_{1}\right)\\ \; & Min\;f_{2}\left(x\right)=g\left(x\right)\left[1-{\left(\frac{f_{1}\left(x\right)}{g(x)}\right)}^{2}\right]\\ \; & g\left(x\right)=1+9{\left[\left(\sum_{i=2}^{n}x_{i}\right)\left(n-1\right)\right]}^{0.25},\\ \; & \mathrm{where}:\;0\leq x\leq 1,n=10. \end{array}$$
